# Palmatine Attenuates LPS-Induced EMT in MAC-T Cells and Mammary Fibrosis in Mice, with Suppression of NF-κB/TGF-β1/Smad Signaling In Vivo

**DOI:** 10.3390/ani16081187

**Published:** 2026-04-14

**Authors:** Dongxue Shi, Dan Bao, Peiru Li, Kejiang Liu, Qi Wang, Weitao Dong, Xingxu Zhao, Yong Zhang

**Affiliations:** 1 College of Veterinary Medicine, Gansu Agriculture University, Lanzhou 730070, China; shidx@gsau.edu.cn (D.S.); d18793272497@163.com (D.B.); 19264348818@163.com (P.L.); lkj1631996@163.com (K.L.); wangqi@gsau.edu.cn (Q.W.); dongwt@gsau.edu.cn (W.D.); 2 Gansu Key Laboratory of Animal Generational Physiology and Reproductive Regulation, Lanzhou 730070, China

**Keywords:** palmatine, bovine mammary epithelial cells, epithelial–mesenchymal transition, mammary fibrosis, NF-κB, TGF-β1/Smad

## Abstract

Bovine mastitis is a common inflammatory disease that can progress to mammary fibrosis, thereby impairing udder health, milk yield, and milk quality. This study investigated the protective effects of palmatine on lipopolysaccharide (LPS)-induced epithelial–mesenchymal transition (EMT) in bovine mammary epithelial cells and mammary fibrosis in mice, as well as the underlying mechanisms. In vitro, palmatine markedly reversed LPS-induced EMT by increasing E-cadherin expression and decreasing N-cadherin and α-SMA expression. In vivo, palmatine alleviated inflammatory cell infiltration and collagen deposition in mammary tissue and reduced the expression of TGF-β1, p-Smad2, p-Smad3, p-p65, TNF-α, IL-1β, and IL-6. These findings suggest that palmatine alleviates LPS-induced mammary fibrosis, possibly through inhibition of the NF-κB/TGF-β1/Smad signaling pathway, and may represent a potential therapeutic strategy for the prevention and treatment of mammary fibrosis.

## Simple Summary

Bovine mastitis is a common disease that can lead to mammary fibrosis, which negatively affects udder health, milk production, and milk quality. In this study, we investigated whether palmatine could protect against lipopolysaccharide (LPS)-induced injury in bovine mammary epithelial cells and mouse mammary tissue. Palmatine reduced epithelial–mesenchymal transition (EMT), inflammation, and collagen deposition. It also suppressed signaling pathways associated with inflammation and fibrosis. These findings suggest that palmatine may be a promising candidate for the prevention and treatment of mammary fibrosis associated with bovine mastitis.

## 1. Introduction

Bovine mastitis is an inflammatory disorder of mammary tissue caused by infection, injury, or other stimuli, among which bacterial infection is the major etiological factor. Lipopolysaccharide (LPS), an important component of the cell wall of Gram-negative bacteria, is a key inducer of severe inflammatory responses and tissue injury in the mammary gland [1]. Under persistent inflammatory stimulation, mammary tissue may further undergo fibrosis, resulting in decreased milk yield and deterioration of milk quality, thereby causing substantial economic losses to the dairy industry [2]. Mammary fibrosis is mainly characterized by excessive extracellular matrix (ECM) deposition, destruction of glandular architecture, and loss of function [3,4,5]. Epithelial–mesenchymal transition (EMT) has been regarded as one of the key events in the development and progression of mammary fibrosis [6]. During this process, epithelial cells progressively lose their polarity and intercellular junctions and acquire mesenchymal-like phenotypes, which is manifested by downregulation of E-cadherin and upregulation of mesenchymal markers such as N-cadherin and α-SMA [7,8,9].

Transforming growth factor-β1 (TGF-β1) is one of the major inducers of EMT [10]. It promotes the recruitment of inflammatory cells and fibroblasts, enhances collagen and fibrin synthesis, and leads to abnormal ECM deposition. Previous studies have shown that TGF-β1 can induce EMT in bovine mammary epithelial cells through activation of the TGF-β1/Smad signaling pathway [11]. Meanwhile, nuclear factor kappa B (NF-κB), a key transcription factor in inflammatory responses, can be activated by LPS and translocated into the nucleus, thereby initiating the transcription of multiple inflammatory cytokines, including TNF-α, IL-1β, and IL-6, and amplifying the local inflammatory response [12]. Persistent inflammatory microenvironments may further promote fibrosis progression. Notably, the NF-κB and TGF-β1/Smad pathways do not function independently but exhibit complex crosstalk. Inflammatory mediators such as TNF-α may promote the synthesis and activation of TGF-β1, whereas NF-κB may also interact with Smad proteins to co-regulate the transcription of fibrosis-related genes, thereby forming a positive feedback loop between inflammation and fibrosis. Therefore, simultaneous targeting of inflammatory and fibrotic signaling pathways may represent an effective strategy for alleviating mammary fibrosis.

Palmatine is a protoberberine-type isoquinoline alkaloid isolated from medicinal plants such as *Fibraurea recisa* and has a wide range of pharmacological activities [13]. Previous studies have shown that palmatine not only exhibits anti-infective potential in bacterial and fungal studies but also reduces the levels of inflammatory mediators such as IL-1β, IL-6, and TNF-α by inhibiting inflammatory signaling pathways including Akt/NF-κB, ERK1/2, p38, and NF-κB/NLRP3 [14,15,16]. Notably, palmatine has been reported to attenuate LPS-induced inflammatory responses in mouse mammary epithelial cells, further supporting its potential protective role in mastitis-related inflammation [16]. In addition, palmatine can alleviate oxidative stress injury by activating the Nrf2/HO-1 pathway [17]. Recent studies have further suggested that palmatine has anti-fibrotic potential, as it can inhibit fibroblast activation, attenuate myocardial fibrosis, and exert protective effects in liver fibrosis models [13,18]. Moreover, palmatine has been shown to reduce mast cell degranulation and abnormal immune-inflammatory responses, indicating that it also possesses immunomodulatory activity [19]. Therefore, palmatine may exert multi-target protective effects in tissue injury-related diseases by simultaneously regulating inflammation, oxidative stress, and fibrosis.

However, the role of palmatine in mammary fibrosis and its underlying mechanisms remains unclear. Therefore, the present study used an in vitro bovine mammary epithelial cell EMT model and an in vivo mouse mammary fibrosis model to evaluate the protective effects of palmatine against LPS-induced mammary fibrosis and to determine whether these effects are associated with inhibition of the NF-κB and TGF-β1/Smad signaling pathways.

## 2. Materials and Methods

### 2.1. Cell Model

MAC-T cells, an immortalized bovine mammary epithelial cell line preserved in our laboratory, were cultured in DMEM/F12 medium (Gibco, Grand Island, NY, USA) supplemented with 10% fetal bovine serum (FBS) at 37 °C in a humidified atmosphere containing 5% CO_2_. To establish a mammary epithelial cell EMT model, MAC-T cells were treated with 25 or 50 μg/mL LPS for 48 h. The cells were then divided into the following groups: control, LPS model, LPS + 20 μg/mL palmatine, LPS + 40 μg/mL palmatine, and LPS + 60 μg/mL palmatine. LPS was purchased from Sigma (Shanghai, China), and palmatine was obtained from Shanghai Yuanye Bio-Technology Co., Ltd. (Shanghai, China).

### 2.2. Establishment of the Animal Model

A total of 24 specific pathogen-free female pregnant Kunming mice were obtained from the Laboratory Animal Center of Lanzhou Veterinary Research Institute, Chinese Academy of Agricultural Sciences. All mice were housed under standard conditions at 23 ± 2 °C, 55 ± 5% relative humidity, and a 12 h light/dark cycle, with free access to food and water. After parturition, the dams were allowed to nurse their pups normally. On lactation day 7, the mice were randomly assigned to four groups (*n* = 6). All mice received intraductal injections into the fourth pair of mammary glands as follows: the Control-7 d group received 50 μL of normal saline; the LPS-7 d group received 50 μL of 200 μg/mL LPS once every 2 d for 7 d; the Control-14 d group received 50 μL of normal saline; and the LPS-14 d group received 50 μL of 200 μg/mL LPS once every 2 d for 14 d. The LPS concentration and injection volume were selected according to a previously published study using a comparable lactating Kunming mouse model of mammary fibrosis [4]. At the end of the experiment, all mice were euthanized by intraperitoneal injection of sodium pentobarbital (150 mg/kg). Mammary tissues were rapidly collected; part of the tissue was fixed in 4% paraformaldehyde, and the remaining tissue was stored at −80 °C for subsequent experiments and model evaluation. The study was carried out in accordance with ethical guidelines that were authorized by Gansu Agricultural University’s Animal Protection Committee (GSAU-Eth-VMC-2021-003).

### 2.3. Palmatine Intervention in the Mouse Mammary Fibrosis Model

Thirty female Kunming mice were randomly divided into a control group, an LPS group, and three palmatine treatment groups receiving low, medium, or high doses (5, 10, and 20 mg/kg, respectively). The source and housing conditions of the mice were the same as described above. All mice received intraductal injections into the fourth pair of mammary glands once every 2 d. The control group received 50 μL of normal saline, whereas the LPS group received 50 μL of 200 μg/mL LPS. Mice in the treatment groups were subjected to LPS challenge, and palmatine treatment was initiated 12 h after the first LPS administration, followed by intraperitoneal injection at the corresponding dose once daily. After 14 d of treatment, all mice were euthanized 12 h after the final administration by intraperitoneal injection of sodium pentobarbital (150 mg/kg). Mammary tissues were rapidly collected; a portion was fixed in 4% paraformaldehyde, and the remainder was stored at −80 °C until use.

### 2.4. Western Blot Analysis

Mouse mammary tissues and MAC-T cells were washed with ice-cold PBS, and total proteins were extracted using RIPA lysis buffer. Equal amounts of protein were separated by SDS-PAGE and transferred onto 0.45 μm PVDF membranes. The membranes were blocked with Tris-buffered saline containing 5% BSA at room temperature for 2 h and then incubated overnight at 4 °C with the following primary antibodies: rabbit polyclonal anti-E-cadherin antibody (20874-1-AP, 1:1000, Proteintech, Wuhan, China), rabbit polyclonal anti-N-cadherin antibody (22018-1-AP, 1:1000, Proteintech, Wuhan, China), rabbit polyclonal anti-α-SMA antibody (14395-1-AP, 1:1000, Proteintech, Wuhan, China), rabbit polyclonal anti-p-p65 antibody (TP56372, 1:1000, Abmart, Shanghai, China), rabbit polyclonal anti-IL-1β antibody (16806-1-AP, 1:1000, Proteintech, Wuhan, China), rabbit polyclonal anti-IL-6 antibody (21865-1-AP, 1:1000, Proteintech, Wuhan, China), mouse monoclonal anti-TNF-α antibody (60291-1-Ig, 1:1000, Proteintech, Wuhan, China), rabbit recombinant monoclonal anti-TGF-β1 antibody (81746-2-RR, 1:1000, Proteintech, Wuhan, China), rabbit polyclonal anti-p-Smad2 antibody (PC3419, 1:1000, Abmart, Shanghai, China), and rabbit polyclonal anti-p-Smad3 antibody (PC5616, 1:1000, Abmart, Shanghai, China). After washing with PBST, the membranes were incubated with HRP-conjugated goat anti-rabbit IgG or goat anti-mouse IgG secondary antibodies (both 1:5000) at 37 °C for 1 h. Protein bands were visualized using ECL chemiluminescence reagent (Vazyme, Nanjing, China) and captured using an Amersham Imager 600 chemiluminescence imaging system (GE Healthcare Bio-Sciences AB, Uppsala, Sweden). Band intensities were analyzed using ImageJ software (version 1.52a), with β-actin used as the internal control.

### 2.5. Immunofluorescence

For immunofluorescence analysis of MAC-T cells, cells grown on coverslips were fixed with 4% paraformaldehyde for 15 min, washed with PBS, and permeabilized with 0.1% Triton X-100 for 10 min. After blocking with 5% BSA at room temperature for 1 h, the cells were incubated overnight at 4 °C with primary antibodies against E-cadherin (1:300), N-cadherin (1:300), and α-SMA (1:300). After washing with PBS, the cells were incubated with fluorescent secondary antibodies. A tri-labeled four-color multiplex fluorescence staining kit (AiFang Biological, Changsha, China) was used for staining, and images were captured using an inverted fluorescence microscope (Revolve Omega, ApexBio, Suzhou, China). For mouse mammary tissue, paraffin sections were subjected to antigen retrieval and immunofluorescence staining following the same procedure. Protein localization and expression were analyzed using ImageJ software (version 1.52a).

### 2.6. Histological Analysis

Mouse mammary tissues were fixed in 4% paraformaldehyde for 24 h, dehydrated routinely, embedded in paraffin, and sectioned at 4 μm thickness. Paraffin sections were subjected to H&E staining and Masson’s trichrome staining to evaluate morphological changes, inflammatory cell infiltration, and collagen deposition in mammary tissue.

### 2.7. Statistical Analysis

Data are presented as the mean ± standard deviation (mean ± SD). Statistical analysis was performed using GraphPad Prism 9 software (GraphPad Software, San Diego, CA, USA). Before performing one-way analysis of variance (ANOVA), homogeneity of variance was assessed using Levene’s test. Comparisons among multiple groups were performed using one-way ANOVA followed by the Student–Newman–Keuls post hoc test. A value of *p* < 0.05 was considered statistically significant. All experiments were performed with three biological replicates (*n* = 3).

## 3. Results

### 3.1. Establishment of an LPS-Induced EMT Model in MAC-T Cells and Identification of the Optimal Concentration of Palmatine

**Figure 1:**
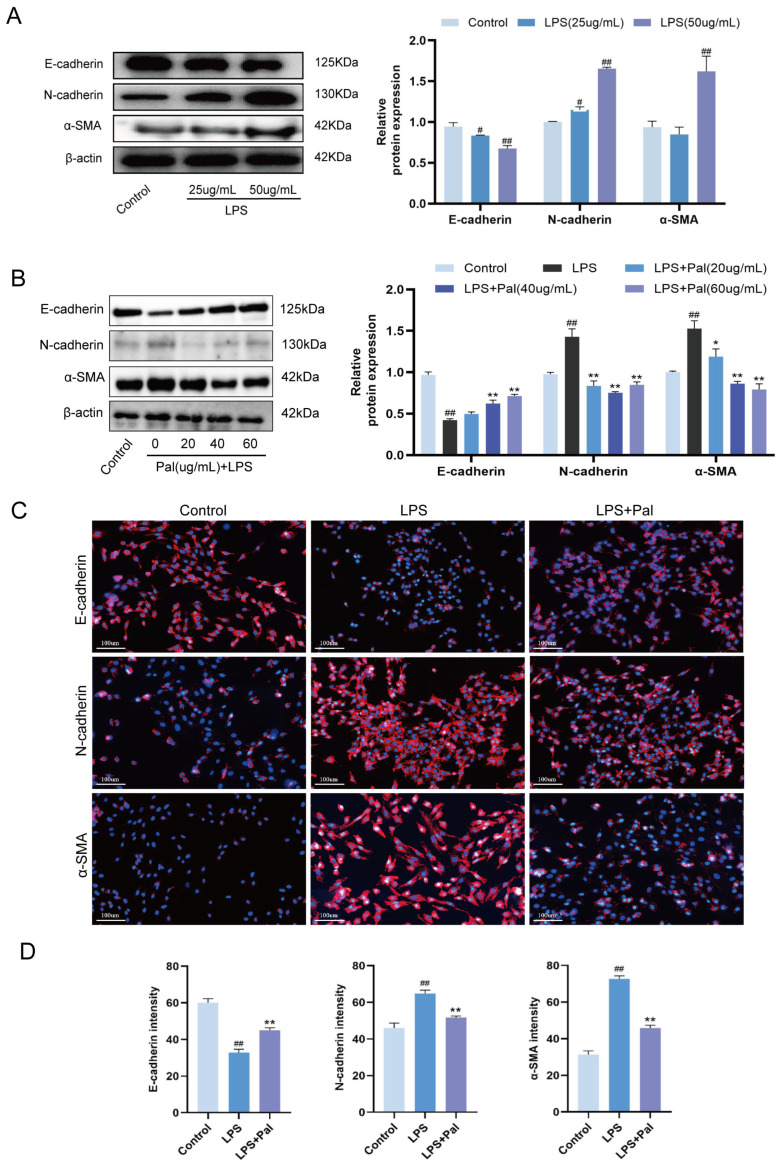
Effects of palmatine on LPS-induced EMT in MAC-T cells. (**A**) MAC-T cells were treated with LPS (25 or 50 μg/mL) for 48 h, and E-cadherin, N-cadherin, and α-SMA expression were detected by Western blotting. (**B**) MAC-T cells were stimulated with 50 μg/mL LPS and treated with different concentrations of palmatine (20, 40, and 60 μg/mL), followed by Western blot analysis of E-cadherin, N-cadherin, and α-SMA expression. (**C**) Immunofluorescence staining of E-cadherin, N-cadherin, and α-SMA in MAC-T cells treated with LPS and 60 μg/mL palmatine. Original magnification: ×400. (**D**) Quantification of fluorescence intensity corresponding to panel (**C**). All experiments were performed in triplicate (*n* = 3). Data are presented as mean ± SD. ^##^ *p* < 0.01, ^#^ *p* < 0.05 vs. the Control group; * *p* < 0.05, ** *p* < 0.01 vs. the LPS group.

To establish an LPS-induced EMT model in MAC-T cells, cells were treated with 25 or 50 μg/mL LPS for 48 h, and the expression of EMT-related proteins was examined. Western blot analysis showed that, compared with the control group, treatment with 50 μg/mL LPS for 48 h significantly decreased the expression of the epithelial marker E-cadherin and significantly increased the expression of the mesenchymal markers N-cadherin and α-SMA (*p* < 0.05) (Figure 1A), indicating that this condition successfully induced EMT in MAC-T cells.

Based on this model, MAC-T cells stimulated with 50 μg/mL LPS were treated with 20, 40, and 60 μg/mL palmatine to determine the optimal concentration of palmatine for inhibiting LPS-induced EMT. Western blot and immunofluorescence results showed that, compared with the control group, LPS stimulation significantly downregulated E-cadherin expression and upregulated N-cadherin and α-SMA expression. Compared with the LPS group, treatment with 60 μg/mL palmatine significantly increased E-cadherin expression and significantly reduced N-cadherin and α-SMA expression (*p* < 0.05) (Figure 1B–D). These findings indicate that 50 μg/mL LPS for 48 h stably induced EMT in MAC-T cells and that 60 μg/mL palmatine exerted a marked inhibitory effect on LPS-induced EMT. These findings indicate that LPS-induced EMT in MAC-T cells provides a cellular model to evaluate the direct effects of LPS and palmatine on mammary epithelial cells. Because epithelial cells are key functional components of mammary tissue, these results suggest that epithelial cell injury and EMT may represent early events in the development of mammary inflammation and fibrosis. Because epithelial cells are key functional components of mammary tissue, these results suggest that mammary epithelial cells are direct responders to LPS stimulation, and that the EMT-related changes observed in vitro may contribute to subsequent fibrotic remodeling under inflammatory conditions.

### 3.2. Establishment of an LPS-Induced Mouse Mammary Fibrosis Model

**Figure 2:**
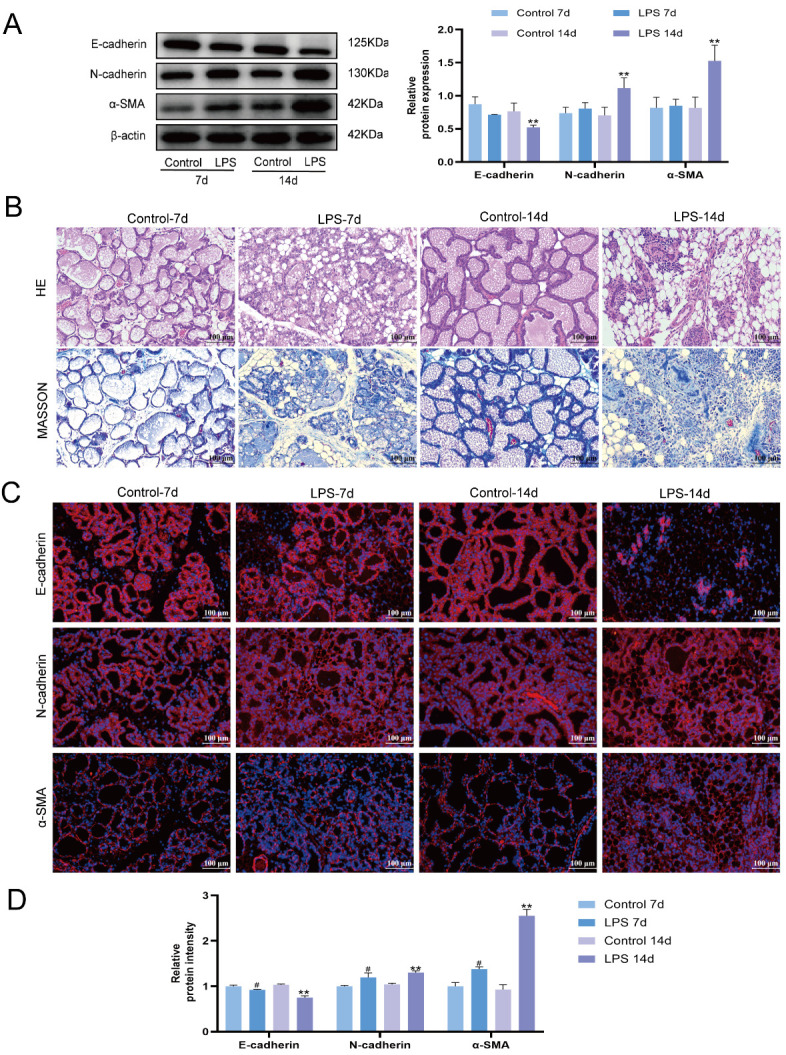
LPS-induced mammary fibrosis model in mice. (**A**) Western blot analysis of E-cadherin, N-cadherin, and α-SMA expression in mammary tissue of mice after 7 and 14 days of LPS stimulation. Mice were stimulated every two days for 7 or 14 days. (**B**) HE staining and Masson’s trichrome staining of mammary tissue sections after 7 and 14 days of LPS stimulation. Original magnification:×200. (**C**) Immunofluorescence analysis of E-cadherin, N-cadherin, and α-SMA expression in mammary tissue sections of mice stimulated with LPS for 7 and 14 days. Original magnification: ×200. (**D**) Quantification of immunofluorescence intensity of E-cadherin, N-cadherin, and α-SMA in mammary tissue sections. All experiments were performed with three independent biological replicates (*n* = 3). Data are presented as mean ± SD. ^#^ *p* < 0.05 vs. the Control-7d group; ** *p* < 0.01 vs. the Control-14d group.

To further determine whether the epithelial alterations observed in vitro are associated with tissue-level pathological changes, an LPS-induced mouse mammary fibrosis model was established. To establish an LPS-induced mouse mammary fibrosis model, mice were subjected to continuous mammary stimulation with LPS for 7 d or 14 d, and the model was comprehensively evaluated by Western blotting, hematoxylin and eosin staining, Masson’s trichrome staining, and immunofluorescence. The results showed that, compared with the control group, E-cadherin protein expression was significantly decreased, whereas N-cadherin and α-SMA expression was significantly increased in mammary tissue after 14 d of LPS stimulation (*p* < 0.05) (Figure 2A). Histological examination showed progressive widening of the mammary interstitium, increasing alveolar injury, aggravated inflammatory cell infiltration, and obvious collagen deposition after prolonged LPS stimulation (Figure 2B). Immunofluorescence further confirmed that E-cadherin expression progressively decreased, whereas N-cadherin and α-SMA expression continuously increased with prolonged LPS stimulation (Figure 2C,D). These results indicate that 14 d of LPS stimulation successfully induced mammary fibrosis in mice.

### 3.3. Identification of the Optimal Dose of Palmatine in the Mouse Mammary Fibrosis Model

**Figure 3:**
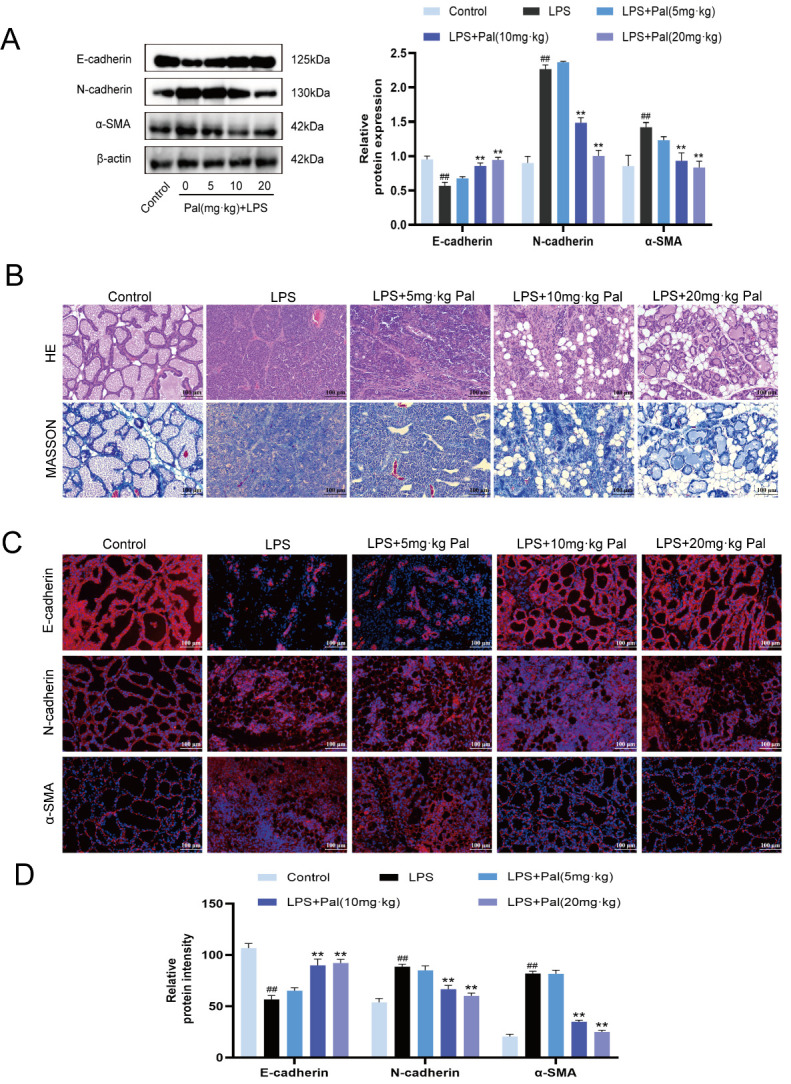
Palmatine attenuates LPS-induced mammary fibrosis in mice. (**A**) Western blot analysis of E-cadherin, N-cadherin, and α-SMA expression in mammary tissue of mice treated with different concentrations of palmatine (5, 10, 20 mg/kg) to alleviate LPS-induced mammary fibrosis. (**B**) HE staining and Masson’s trichrome staining of mammary tissue sections from mice treated with palmatine at various concentrations. Original magnification: ×200. (**C**) Immunofluorescence detection of E-cadherin, N-cadherin, and α-SMA expression in mammary tissue sections from mice treated with palmatine. Original magnification: ×200. (**D**) Quantification of immunofluorescence intensity of E-cadherin, N-cadherin, and α-SMA in mammary tissue sections. All experiments were performed with three independent biological replicates (*n* = 3). Data are presented as mean ± SD. ^##^ *p* < 0.01 vs. the Control group; ** *p* < 0.01 vs. the LPS group.

To determine the optimal dose of palmatine for alleviating mammary fibrosis, different doses of palmatine (5, 10, and 20 mg/kg) were administered 12 h after the first LPS challenge and continued during the establishment of the LPS-induced mouse mammary fibrosis model. Compared with the LPS group, treatment with 20 mg/kg palmatine significantly increased the protein expression of E-cadherin and significantly decreased the expression of N-cadherin and α-SMA in mammary tissues (*p* < 0.05) (Figure 3A). Hematoxylin and eosin staining suggested that palmatine reduced inflammatory cell infiltration and improved alveolar structural injury. Masson’s trichrome staining indicated a decrease in collagen deposition in mammary tissue (Figure 3B). In addition, immunofluorescence analysis confirmed that palmatine enhanced E-cadherin fluorescence intensity while reducing the fluorescence intensity of N-cadherin and α-SMA (Figure 3C,D). These results suggest that 20 mg/kg palmatine effectively alleviated LPS-induced mammary fibrosis.

### 3.4. Palmatine Alleviated Mouse Mammary Fibrosis by Inhibiting the NF-κB and TGF-β1/Smad Signaling Pathways

**Figure 4:**
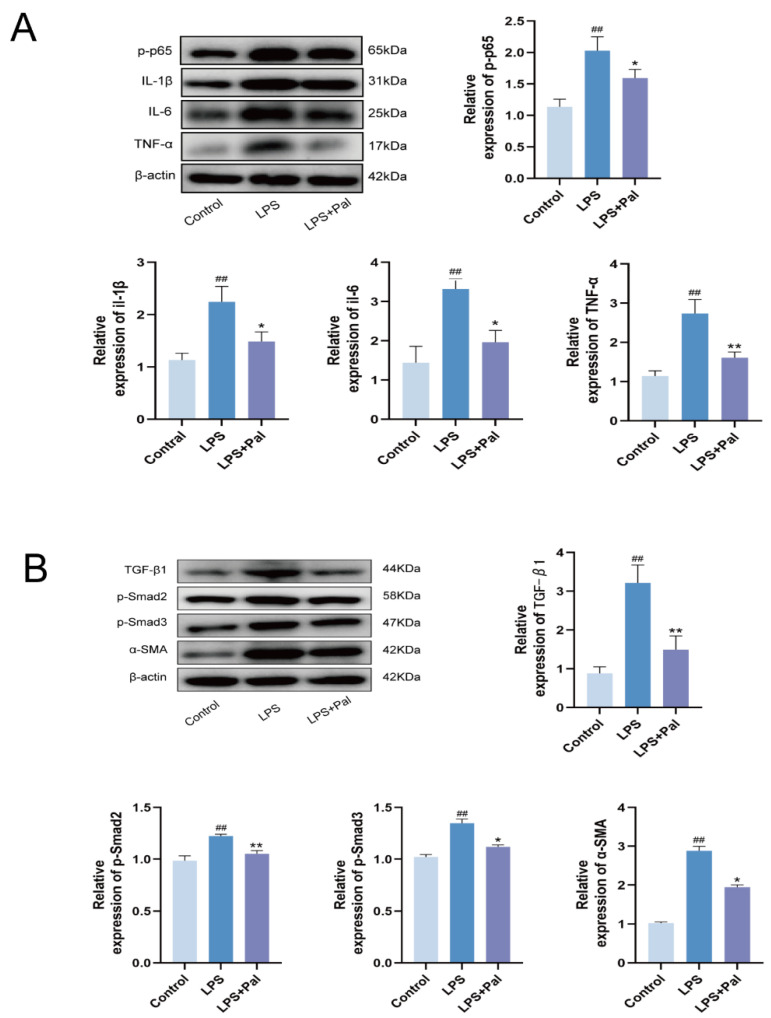
Palmatine attenuates LPS-induced mammary inflammation and fibrosis in mice by inhibiting the NF-κB and TGF-β1/Smad signaling pathways. (**A**) Western blot analysis of p-p65, IL-1β, IL-6, and TNF-α expression in mammary tissue of mice treated with palmatine to assess the inhibition of the NF-κB signaling pathway. (**B**) Western blot analysis of TGF-β1, p-Smad2, p-Smad3, and α-SMA expression in mammary tissue of mice treated with palmatine to evaluate the suppression of the TGF-β1/Smad signaling pathway. All experiments were performed with three independent biological replicates (*n* = 3). Data are presented as mean ± SD.^##^ *p* < 0.01 vs. the Control group; * *p* < 0.05, ** *p* < 0.01 vs. the LPS group.

Based on the above findings that LPS induced EMT and epithelial injury both in vitro and in vivo, we further investigated whether these changes were associated with activation of key inflammatory and fibrotic signaling pathways in mammary tissue. To further explore the molecular mechanism by which palmatine alleviated mammary fibrosis, mice were divided into control, LPS, and LPS + palmatine groups, and the expression of proteins related to the NF-κB and TGF-β1/Smad signaling pathways was analyzed by Western blotting. Compared with the control group, the LPS group showed significantly increased expression of p-p65, IL-1β, IL-6, and TNF-α (*p* < 0.05), whereas palmatine treatment significantly reduced the expression of these proteins compared with the LPS group (*p* < 0.05) (Figure 4A), suggesting that palmatine suppressed LPS-induced activation of the NF-κB signaling pathway and the associated inflammatory response.

Further analysis of TGF-β1/Smad pathway-related proteins showed that, compared with the control group, the LPS group exhibited significantly increased expression of TGF-β1, p-Smad2, p-Smad3, and α-SMA (*p* < 0.05). In contrast, palmatine treatment significantly reduced the expression of these proteins compared with the LPS group (*p* < 0.05) (Figure 4B). These findings indicate that palmatine alleviated LPS-induced mammary inflammatory and fibrotic injury in mice by simultaneously suppressing activation of the NF-κB and TGF-β1/Smad2/3 signaling pathways.

## 4. Discussion

Mammary epithelial cells are the major functional cells in mammary tissue. EMT is also recognized as an important event in fibrosis. Therefore, we established an in vitro mammary epithelial cell EMT model to evaluate epithelial phenotypic transition under inflammatory stimulation. LPS was used as the inducer because it is a major pro-inflammatory component of Gram-negative bacteria and a common trigger of inflammatory injury and fibrotic remodeling [20,21,22,23]. In MAC-T cells, LPS decreased E-cadherin expression and increased N-cadherin and α-SMA expression, indicating successful induction of EMT.

To further determine whether the epithelial alterations observed in vitro were associated with more integrated pathological changes in vivo, we established an LPS-induced mouse mammary fibrosis model. In mammary tissue, prolonged LPS stimulation also reduced E-cadherin expression and increased N-cadherin and α-SMA expression, accompanied by inflammatory cell infiltration, alveolar structural damage, and marked collagen deposition. These findings suggest that the epithelial phenotypic transition observed in vitro is associated with tissue-level inflammatory injury and fibrotic remodeling in vivo. Taken together, the in vitro and in vivo results support the idea that persistent LPS stimulation promotes mammary fibrosis, at least in part, through epithelial injury and EMT-associated changes.

Palmatine has been reported to possess anti-inflammatory, antioxidant, and anti-fibrotic activities [24,25]. However, its effects on mammary fibrosis have not been well characterized. In the present study, palmatine treatment significantly increased E-cadherin expression and decreased N-cadherin and α-SMA expression in LPS-stimulated MAC-T cells, indicating that palmatine effectively inhibited LPS-induced EMT in mammary epithelial cells. In the mouse model, palmatine also alleviated mammary histopathological injury, reduced inflammatory cell infiltration and collagen deposition, and reversed the abnormal expression of EMT-related proteins. These findings consistently indicate that palmatine exerts protective effects against LPS-induced mammary injury and fibrosis both at the cellular and tissue levels.

An important issue raised by the present results is the relationship between the in vitro MAC-T cell model and the in vivo mouse model. These two experimental systems were designed to address different but complementary levels of the proposed mechanism. The MAC-T cell model was used to determine whether LPS directly acts on mammary epithelial cells and induces EMT-related changes, and whether palmatine can counteract these direct cellular effects. In contrast, the mouse mammary fibrosis model was used to validate whether these early cellular alterations are accompanied by inflammatory activation, fibrotic signaling, and pathological remodeling at the tissue level. Since mammary inflammation and fibrosis are complex biological processes involving multiple cell types, cytokine networks, extracellular matrix metabolism, and microenvironmental interactions, they cannot be fully recapitulated in a single-cell in vitro system. Therefore, the MAC-T experiments should be regarded as mechanistic support at the cellular level, whereas the mouse model provides pathophysiological validation in vivo.

The TGF-β1/Smad signaling pathway is a classical pathway involved in EMT regulation and tissue fibrosis [26,27]. TGF-β1 promotes myofibroblast activation, ECM deposition, and fibrosis progression mainly through phosphorylation of Smad2 and Smad3 [28,29,30,31]. In the present study, LPS significantly increased the expression of TGF-β1, p-Smad2, p-Smad3, and α-SMA in mouse mammary tissue, while palmatine markedly reduced these changes. These results suggest that activation of the TGF-β1/Smad pathway is involved in LPS-induced mammary fibrotic remodeling and that palmatine may attenuate fibrosis by suppressing this pathway.

In addition to fibrotic signaling, NF-κB plays a central role in inflammatory responses. Activation of NF-κB promotes the production of inflammatory cytokines such as IL-1β, IL-6, and TNF-α, thereby amplifying local inflammation and creating a microenvironment favorable for fibrosis progression [32,33]. In this study, LPS significantly increased the expression of p-p65, IL-1β, IL-6, and TNF-α in mouse mammary tissue, whereas palmatine significantly reduced these inflammatory markers, indicating that palmatine suppressed LPS-induced inflammatory activation through inhibition of the NF-κB pathway. Moreover, NF-κB-mediated inflammation and TGF-β1/Smad-mediated fibrosis are closely interconnected, and their interaction may further promote EMT, collagen synthesis, and tissue remodeling. Therefore, the protective effect of palmatine against mammary fibrosis may be associated with simultaneous suppression of inflammatory and fibrotic signaling.

It should be noted that NF-κB and TGF-β1/Smad signaling pathways were validated in mouse mammary tissue but were not directly examined in MAC-T cells in the present study. Thus, the in vitro findings should be interpreted as supportive evidence showing that mammary epithelial cells are directly involved in the early response to LPS, rather than as direct validation of these signaling pathways at the cellular level. Future studies should further investigate whether palmatine directly regulates NF-κB and TGF-β1/Smad signaling in MAC-T cells and should include pathway-specific inhibition or rescue experiments to better define the molecular targets of palmatine.

**Figure 5:**
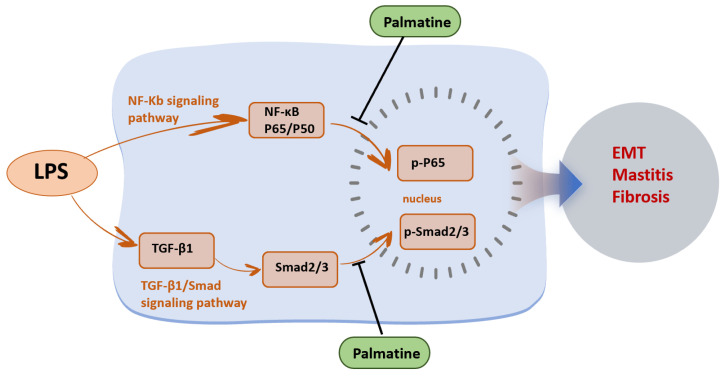
Palmatine inhibits mammary fibrosis by suppressing the NF-κB and TGF-β1/Smad signaling pathways.

In summary, our findings suggest that palmatine alleviates LPS-induced mammary injury and fibrosis by inhibiting epithelial EMT-associated changes, suppressing NF-κB-mediated inflammatory responses, and attenuating TGF-β1/Smad-mediated fibrotic signaling (Figure 5). These results provide experimental evidence supporting the potential application of palmatine in the prevention or treatment of inflammation-associated mammary fibrosis. Nevertheless, because the in vivo experiments were conducted in mice rather than dairy cows, further validation in bovine mammary disease models is still needed before clinical application.

## 5. Conclusions

In conclusion, palmatine markedly alleviated LPS-induced EMT in MAC-T cells and mammary fibrosis in mice. The protective effects of palmatine were closely associated with inhibition of the NF-κB and TGF-β1/Smad signaling pathways. Palmatine may therefore represent a promising candidate for the prevention and treatment of bovine mastitis and mammary fibrosis.

## Data Availability

The original contributions presented in this study are included in the article. Further inquiries can be directed to the corresponding authors.
